# CD86^+^/CD206^+^ tumor-associated macrophages predict prognosis of patients with intrahepatic cholangiocarcinoma

**DOI:** 10.7717/peerj.8458

**Published:** 2020-01-22

**Authors:** Dalong Sun, Tiancheng Luo, Pingping Dong, Ningping Zhang, Jing Chen, Shuncai Zhang, Longzi Liu, Ling Dong, Si Zhang

**Affiliations:** 1 Department of Gastroenterology and Hepatology, Zhongshan Hospital, Fudan University, Shanghai, China; 2 Department of Gastroenterology and Hepatology, Xiamen Branch, Zhongshan Hospital, Fudan University, Xiamen, China; 3 Shanghai Institute of Liver Disease, Shanghai, China; 4 Department of Surgery, Faculty of Medicine, The University of Hong Kong, Hong Kong, China; 5 Department of Neurology, Zhongshan Hospital, Fudan University, Shanghai, China; 6 Department of General Surgery, The First Affliated Hospital of Nanchang University, Nanchang, Jiangxi, China; 7 NHC Key Laboratory of Glycoconjugate Research, Department of Biochemistry and Molecular Biology, Shanghai Medical College, Fudan University, Shanghai, China

**Keywords:** Intrahepatic cholangiocarcinoma, Tumor-associated macrophages, CA-199, Prognosis, CD206, CD86

## Abstract

**Background:**

As the main cellular ingredients of tumor microenvironment, tumor-associated macrophages (TAMs) play a vital role in tumor development and progression. Recent studies have suggested that TAMs are sensitive and specific prognostic factors in numerous cancers. The primary purpose of this study is to determine the prognostic significance of TAMs in intrahepatic cholangiocarcinoma (ICC).

**Methods:**

Immunohistochemical staining of CD68, CD86 and CD206 were performed in tissue microarrays containing 322 patients, who underwent surgical resection and were pathologically diagnosed with ICC. The prognostic value of CD68, CD86 and CD206 were evaluated by Kaplan–Meier analysis (log-rank test) and nomogram models.

**Results:**

We demonstrated that the CD86^+^/CD206^+^ TAMs model was an independent prognostic index for ICC patients. Patients with low CD86^+^ TAMs and high CD206^+^ TAMs infiltration had a markedly worse prognosis and increased risk of post-operative recurrence when compared to high CD86^+^ TAMs and low CD206^+^ TAMs intratumoral infiltration. Furthermore, subgroup analysis indicated that the CD86^+^/CD206^+^ TAMs model predicted prognosis of ICC patients more powerfully than single macrophage immunomarker. Interestingly, the CD86^+^/CD206^+^ TAMs model could further distinguish prognosis of CA-199 negative ICC patients, who were generally presumed to have a more favorable outcome. In order to further perfect the prognostic value of the CD86^+^/CD206^+^ TAMs model, we constructed and validated a postoperative nomogram to predict overall survival and recurrence-free survival time in ICC patients.

**Conclusions:**

These findings indicate that the CD86^+^/CD206^+^ TAMs model possess potential value as a novel prognostic indicator for ICC patients.

## Introduction

Intrahepatic cholangiocarcinoma (ICC), derived from the intrahepatic biliary tree, ranks second in the morbidity of liver malignancies (Blechacz & Gores, 2008). Despite of the application of systemic therapy and novel targeted drugs, the prognosis of ICC patients remains poor, especially in advanced cases (Shaib et al., 2004). Complete resection of the tumor lesion is the only potential curative option, but postoperative tumor recurrence or metastasis is inevitable, with a median overall survival of approximately 30 months (Farges et al., 2011; De Jong et al., 2011). However, due to genetic complexity and tumor heterogeneity, the clinical outcomes of patients with parallel clinical and pathological characteristics may vary significantly. Current prognostic models based on integrated clinicopathologic features, such as carbohydrate antigen 199 (CA-199), lymph node metastasis and tumor-node-metastasis (TNM) stage, may not be sufficient in predicting clinical outcomes of ICC patients. A more accurate prognostic model is warranted to be established to better predict clinical outcomes of ICC patients.

Accumulated studies have demonstrated that the tumor microenvironment is critical for tumor development and progression (Liotta & Kohn, 2001). Macrophage is one of the various cellular ingredients involved in the formation of tumor microenvironment, in which they are commonly known as tumor-associated macrophages (TAMs). In response to different microenvironment signals, macrophages can alter their expression profile and transform into different phenotypes including M1 and M2 macrophages (Fridman et al., 2012; Murray & Wynn, 2011). M1 macrophages (“killing” phenotype) are activated by toll-like receptor ligands and interferon-γ (IFN-γ), exhibiting anti-tumor properties. M1 macrophages also amplify Th1 responses, providing a positive feedback loop in anti-tumor response, while M2 macrophages (“healing” phenotype) are mainly stimulated by interleukin-4 (IL-4) or IL-13, inclined to facilitate tumor growth and progression (Murray & Wynn, 2011; Shi et al., 2015; Martinez & Gordon, 2014; Solinas et al., 2009; Mantovani et al., 2002).

CD68 is the most commonly used marker for the study of TAMs, but it is not a specific marker of macrophages and cannot effectively distinguish M1 and M2 subtype macrophages (Falini et al., 1993). In recent years, compelling evidence showed that M1 subtype macrophages expressed abundant CD86, CD38, Gpr18, Fpr2 and tumor necrosis factor α, whereas M2 subtype macrophages expressed high levels of CD206, CD163, Egr2 and c-Myc (Biswas & Mantovani, 2010; Olsson et al., 2015; Jablonski et al., 2015). Recent studies found that M1 subtype macrophages possessed high levels of CD86, while M2 subtype macrophages expressed high levels of CD206 in human gastrointestinal tumors tissues (Hernandez et al., 2014; Zhu et al., 2015).

Our previous study reported that low presence of CD86^+^ M1 TAMs and high presence of CD206^+^ M2 TAMs were significantly correlated with aggressive tumor phenotypes and worse prognosis in HCC patients (Dong et al., 2016). However, the profile of TAMs alteration and its correlation with ICC prognosis remain uncertain. In this study, we investigated the clinical relevance and prognostic significance of CD86^+^/CD206^+^ TAMs in patients diagnosed with ICC.

## Materials and Methods

### Clinical patients

A total of 322 ICC patients who underwent surgical resection and were pathologically diagnosed with ICC at Zhongshan Hospital, Fudan University (Shanghai, China) between May 2005 and April 2006 were enrolled in the study (Liu et al., 2016). Post-operative follow-up was consistent with harmonized standard. The clinicopathological and baseline demographic characteristics of the patients were retrospectively collected. OS was defined as the time frame from the date of operation to death, and recurrence-free survival time (RFS) was defined as the time interval from the date of surgery to recurrence (Dong et al., 2016). All patients were followed until recurrence of disease, death or lost to follow-up. This study was approved by the Clinical Research Ethics Committee of Zhongshan Hospital (No. y2017-179) and all patients provided written informed consent prior to the study. The consent inform is mainly concerned with informing patients of collecting their clinicopathological data without patient privacy exposure and obtaining biological samples for protein or genetic testing.

### Immunohistochemistry

Protocols and details of tissue microarray (TMA) sections and immunohistochemistry (IHC) were performed as previously described (Liu et al., 2016). TMAs are produced using formalin-fixed paraffin-embedded (FFPE) ICC tissue samples. Representative regions were premarked in the paraffin-embedded tumor tissue. Sample cores were selected and taken from each representative ICC tissue. Serial sections (4 μm thick) were placed on slides coated with 3-aminopropyltriethoxysilane. IHC was performed using a two-step protocol. Paraffin sections were first deparaffinized and hydrated. After microwave antigen retrieval and neutralization of endogenous peroxidase, slides were preincubated with blocking serum and then incubated overnight with primary antibodies (anti-CD68 antibody, anti-CD86 antibody, Abcam; anti-CD206 antibody, Abcam; anti-CD31 antibody, Abcam; anti- LYVE1 antibody, diluted at 1:100, Abcam). Subsequently, the sections were serially rinsed, incubated with second antibodies. Positive staining was visualized with DAB (3, 3-diaminobenzidine), then counterstained with hematoxylin. The IHC staining of the TMA sections was evaluated manually under the microscope (DP73; Olympus, Tokyo, Japan). The positive staining cells were counted by Image Pro Plus 6.0 analysis software (Media Cybernetics, Bethesda, MD, USA) and the detailed procedure is described elsewhere (Brey et al., 2003).The positive staining TAMs cells were identified as brown colored, irrespective of color depth, and were counted in five randomly selected visual fields under high magnification (×200). Representative low or high immunostaining density of CD68^+^, CD86^+^ and CD206^+^ TAMs was recorded. The immunohistochemical staining was evaluated independently by two experienced researchers blinded to the patients’ clinical characteristics and outcomes. The intra-observer reproducibility was tested by obtaining statistical κ-scores (Landis & Koch, 1977). Based on the median values of CD68, CD86 and CD206 positive TAMs, the ICC patients were classified into high and low subgroups (Kuang et al., 2011).

### Multiple immunofluorescence labeling of formalin-fixed paraffin-embedded tissue

Multiple immunofluorescence labeling of FFPE tissue was performed as mentioned previously (Robertson & Isacke, 2011). In brief, slides were dewaxed and rehydrated. Then transfer the slide rack into prewarmed target retrieval solution. After rinsed with PBS and immunofluorescence buffer, the samples were incubated with prediluted primary antibody, then washed with 1× PBS and incubated with corresponding secondary horseradish peroxidase-conjugated polymer for antibody conjugation. Fluorescein tyramide signal amplification plus (PerkinElmer, Waltham, MA, USA) was used for amplifying signals. After serials rinsing, slides were placed in specific retrieval buffer in microwave to remove redundant antibodies before the sequenced staining. Nuclei were counterstained with 4′-6-diamidino-2-phenylindole (DAPI) for 5 min. All slides were examined under a laser confocal microscope (Leica TCS SP5; Leica, Wetzler, Germany).

### Statistical analysis

Statistical analyses were performed using SPSS software (20.0; SPSS, Inc., Chicago, IL, USA) and R software version 3.3.2. Univariate predictors of the status of immunohistochemical biomarkers (CD68, CD86 and CD206) were evaluated using the Pearson χ^2^ test. OS and RFS curves were depicted using Kaplan–Meier method and compared with log-rank test. A Cox proportional hazards model was used to identify potential predictors and to assess the relationship between immunohistochemical biomarkers and survival. The statistically significant prognostic indicators for OS and RFS were selected to build nomogram models, using the “rms” package (R Foundation for Statistical Computing, Vienna, Austria). Furthermore, calibration plots and area under the receiver operating characteristic (ROC) curves (AUC) were used to evaluate the performance of the nomograms with “pROC” package (R Foundation for Statistical Computing, Vienna, Austria). A two-sided value of *p* < 0.05 was considered statistically significant.

## Results

### Immunohistochemical and immunofluorescence characterizations of tumor-associated macrophages in ICC Patients

**Figure 1:**
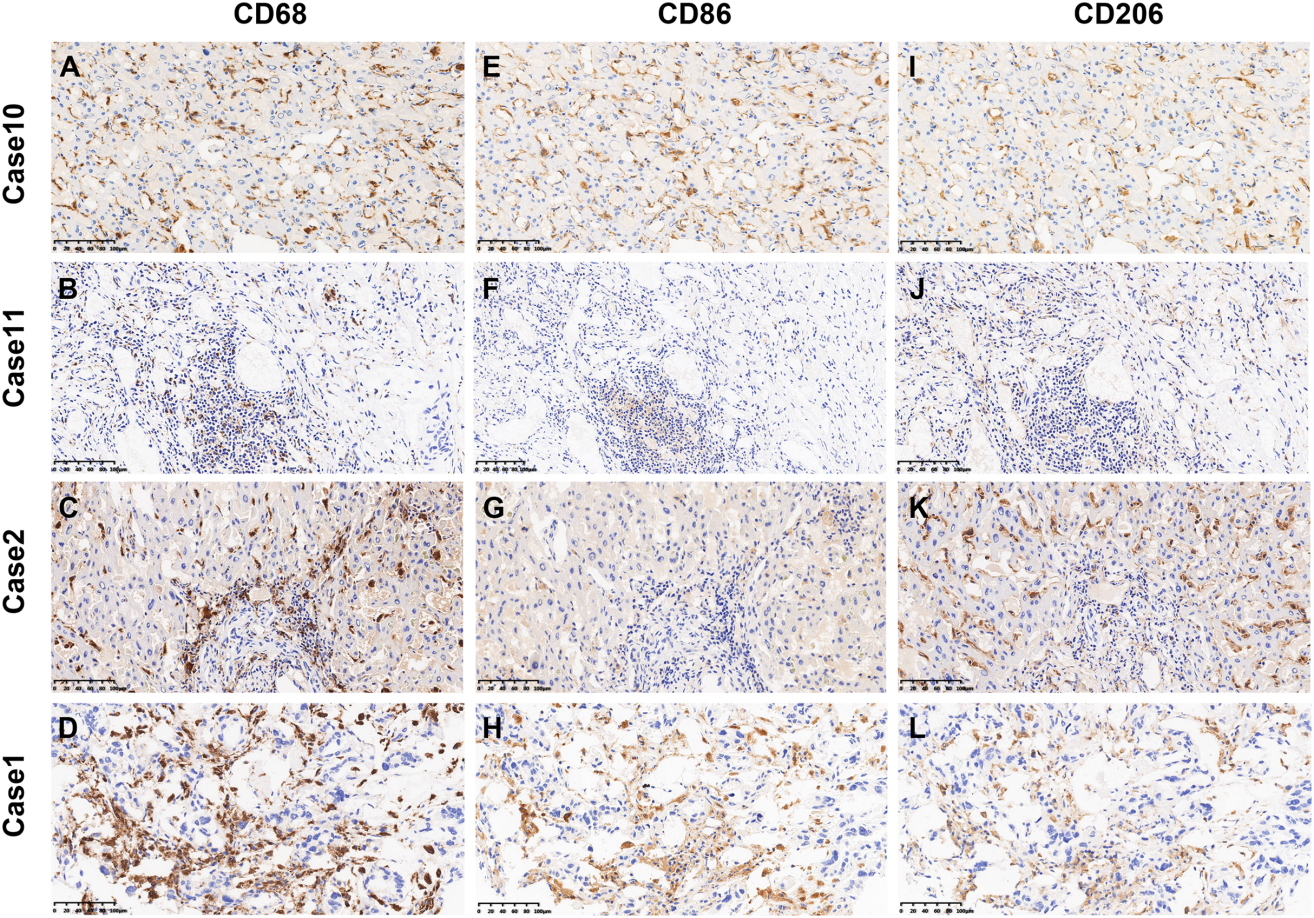
Representative images of CD68^+^, CD86^+^ and CD206^+^ immunostaining in ICC. (A–D) The representative photographs of CD68^+^ TAMs. (E–H) The representative photographs of CD86^+^ TAMs. (I–L) The representative photographs of CD206^+^ TAMs. Patient 67 (A, E and I) showed high immunostaining density of CD68^+^, CD86^+^ and CD206^+^ TAMs. Patient 202 (B, F and J) showed low immunostaining density of CD68^+^, CD86^+^ and CD206^+^ TAMs. Patient 12 (C, G and K) showed high immunostaining density of CD68^+^ and CD206^+^ TAMs and low immunostaining density of CD86^+^ TAMs. Patient 145 (D, H and L) showed high immunostaining density of CD68^+^ and CD86^+^ TAMs and low immunostaining density of CD206^+^ TAMs. Magnification: ×200.

**Figure 2:**
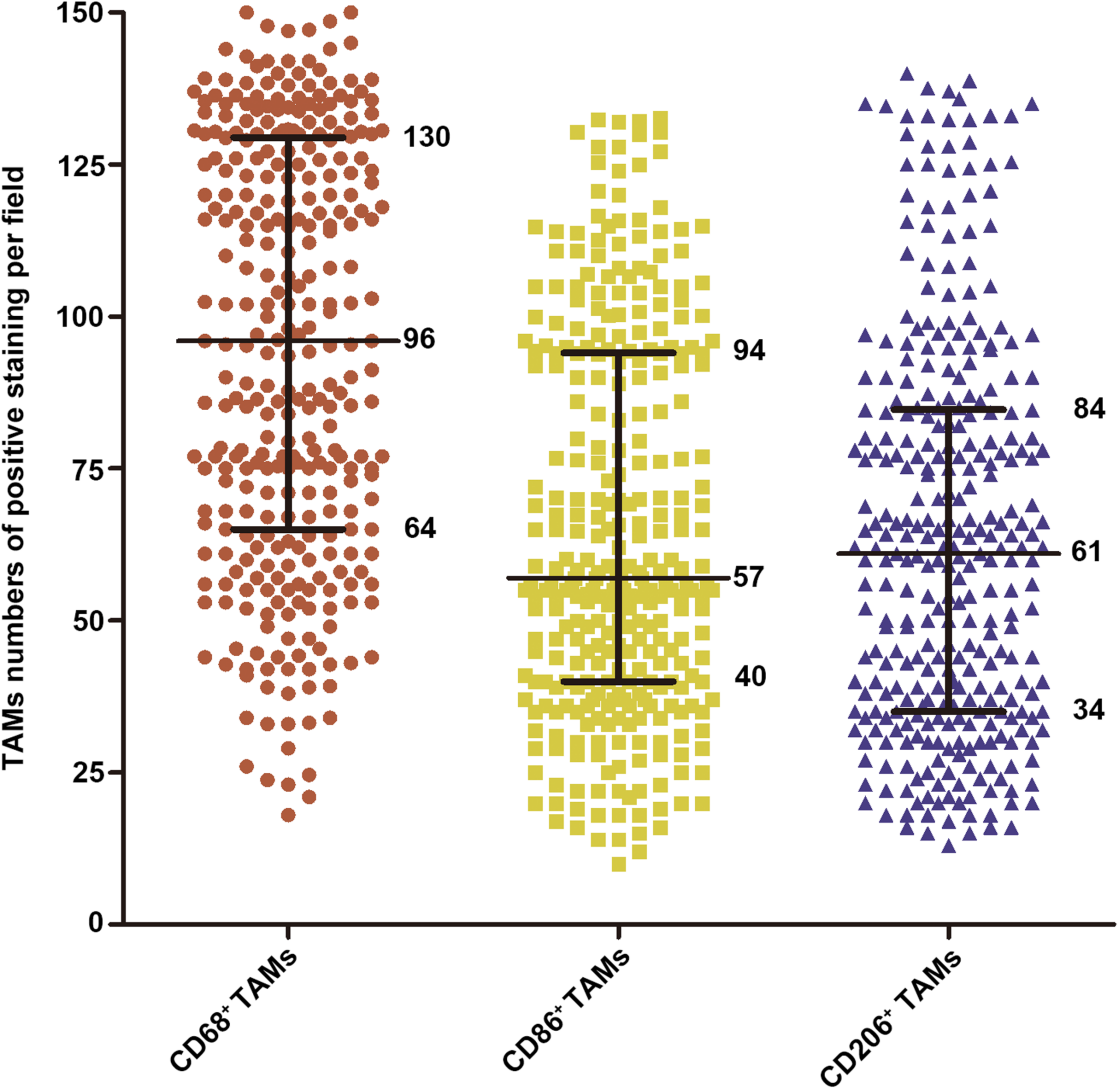
The density distribution of CD68^+^, CD86^+^ and CD206^+^ TAMs in 322 ICC patients. The 25th, 50th and 75th percentiles were labeled.

As shown in Fig. 1, the positive staining of CD68, CD86 and CD206 were observed mostly in the cytoplasm of TAMs. Figure S1 showed the immunofluorescence staining and the colocalization of CD68^+^, CD86^+^ and CD206^+^ macrophages in the same image. The average levels of CD68 positive staining cells (median, 96 cells/field) was higher than that of CD86 positive staining cells (median, 57 cells/field) and CD206 positive staining cells (median, 61 cells/field, Fig. 2).

### Correlations between intratumoral expression of TAMs and clinicopathologic characteristics in ICC patients

Based on the immunohistochemical findings of TAMs in 322 ICC patients, the potential correlation between TAMs and patients’ clinical characteristics was evaluated. The associations were shown in Table 1. CD68^+^ TAMs had no association with patients’ clinicopathologic features. Low intratumoral infiltration of CD86^+^ TAMs positively correlated with higher preoperative CA-199 (*p* = 0.014), appearance of lymph node metastasis (*p* = 0.012), presence of liver cirrhosis (*p* = 0.008) and advanced TNM staging (*p* = 0.046); while high CD206^+^ TAMs infiltration was significantly associated with presence of lymph node metastasis (*p* = 0.005), vascular invasion (*p* = 0.038) and high-grade TNM staging (*p* = 0.001). To investigate the relationship among tumorinfiltrating M2 macrophages and angiogenesis and lymph node metastasis, IHC experiments were performed for the evaluation of microvessel density (MVD) and lymphatic microvessel density (LMVD) via staining of CD31 and LYVE-1 in 15 samples of both groups. As shown in Fig. S2A, Case 4 represented a sample with high density of M2-TAMs, MVD and LMVD while case 11 showed a sample with low density of M2-TAMs, MVD and LMVD. It revealed that the MVD was significantly higher in the CD206 high tissues compared with the CD206 low tissues (10.76 ± 0.66 vs. 7.47 ± 0.40, *p* = 0.0002), which indicated that M2 macrophages may stimulate angiogenesis (Fig. S2B). But there is no significant difference between the two groups in LMVD (*p* > 0.05) (Fig. S2C).

**Table 1:** Correlation between immunohistochemical variables and clinicopathologic features of ICC patients in the cohort (*n* = 322).

Characteristics	CD68^+^ TAMs			CD86^+^ TAMs			CD206^+^ TAMs		
	Low	High	*P*^#^	Low	High	*P*^#^	Low	High	*P*^#^
Age, years									
≤51	88	81	0.503	87	82	0.655	79	90	0.264
>51	73	80		74	79		82	71	
Gender									
Female	69	59	0.305	57	71	0.139	71	57	0.139
Male	92	102		104	90		90	104	
Hepatitis history									
No	104	94	0.303	94	104	0.303	100	98	0.909
Yes	57	67		67	57		61	63	
CA199 (U/ml)									
≤36	85	72	0.181	67	90	**0.014**	86	71	0.118
>36	76	89		94	71		75	90	
Lymph node metastasis									
No	135	131	0.659	124	142	**0.012**	143	123	**0.005**
Yes	26	30		37	19		18	38	
Liver cirrhosis									
No	121	115	0.529	107	129	**0.008**	126	110	0.058
Yes	40	46		54	32		35	51	
Tumor size (cm)									
≤5	75	70	0.654	75	70	0.654	80	65	0.117
>5	86	91		86	91		81	96	
Tumor number									
Single	121	123	0.897	120	124	0.697	123	121	0.897
Multiple	40	38		41	37		38	40	
Vascular invasion									
No	135	141	0.426	133	143	0.151	145	131	**0.038**
Yes	26	20		28	18		16	30	
Tumor encapsulation									
None	146	137	0.171	145	138	0.305	141	142	1.000
Complete	15	24		16	23		20	19	
Tumor differentiation									
I–II	127	122	0.595	122	127	0.595	127	122	0.595
III	34	39		39	34		34	39	
TNM stage									
I + II	129	119	0.233	116	132	**0.046**	137	111	**0.001**
III + IV	32	42		45	29		24	50	

**Notes:**

ICC, intrahepatic cholangiocarcinoma; TNM, tumor-node-metastasis.

Bold values indicate *P* < 0.05.

# The Pearson Chi square test was applied.

### Survival analysis with a single macrophages immunomarker (CD68, CD86 and CD206) in ICC patients

**Figure 3:**
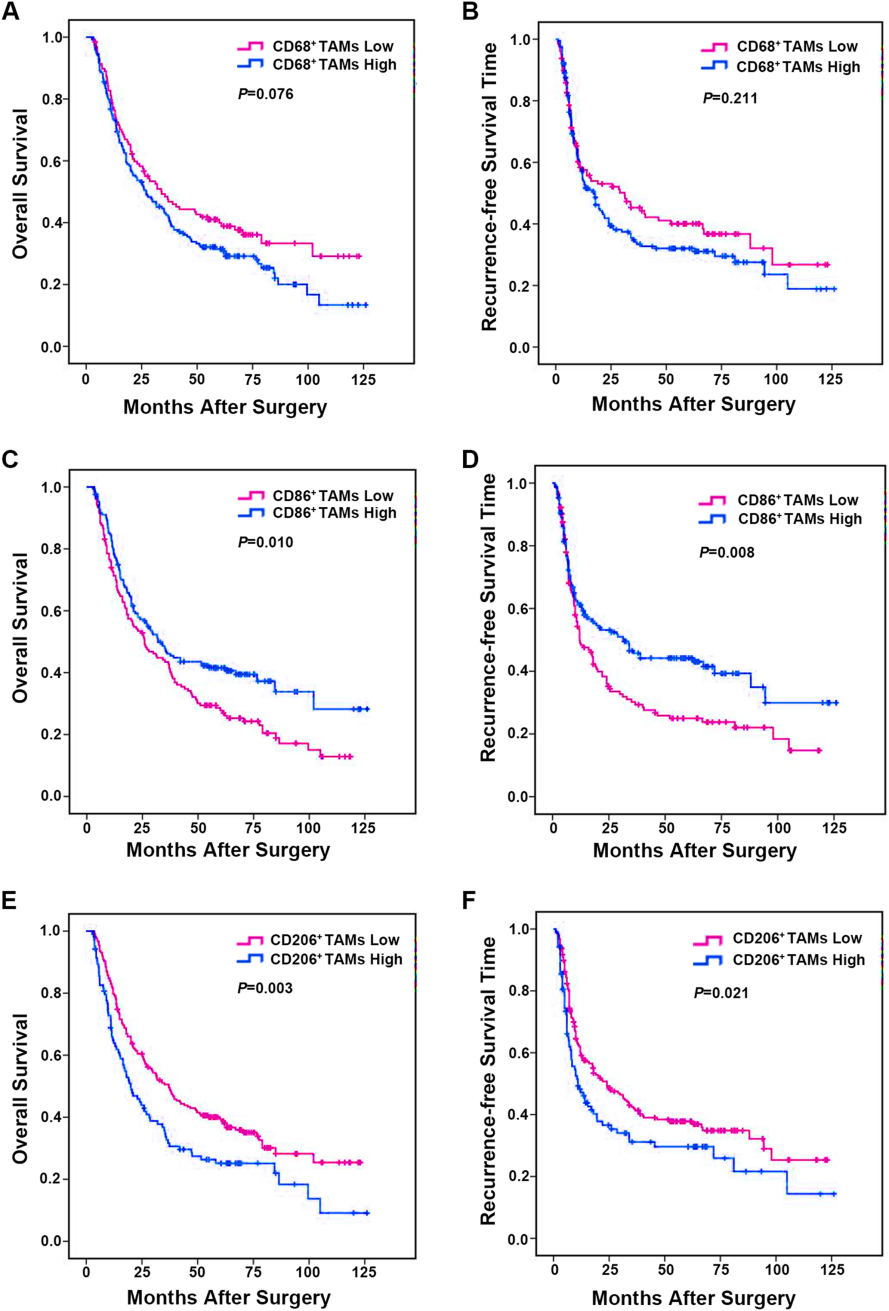
Kaplan–Meier analysis of OS and RFS in ICC based on the immunostaining density of TAMs. (A) OS for CD68^+^ TAMs. (B) RFS for CD68^+^ TAMs. (C) OS for CD86^+^ TAMs. (D) RFS for CD86^+^ TAMs. (E) OS for CD206^+^ TAMs and (F) RFS for CD206^+^ TAMs. *p* Values were calculated by log-rank test, and *p* < 0.05 was considered statistically significant. OS, Overall survival; RFS, recurrence-free survival.

The prognostic value of single macrophages immune marker (CD68, CD86 and CD206) in this cohort of 322 ICC patients was further investigated. The result demonstrated that, as a single variable, CD68^+^ TAMs density had no prognostic value (Figs. 3A and 3B). Patients with low CD86^+^ TAMs infiltration had a markedly worse median overall OS and shorter RFS (OS, 42.6 months; RFS, 37.0 months) when compared to those with high CD86^+^ TAMs expression (OS, 58.2 months, *p* = 0.010; RFS, 56.1 months, *p* = 0.008) (Figs. 3C and 3D). In contrast, patients with low CD206^+^ TAMs presence had a longer median OS and RFS (OS, 55.1 months; RFS, 50.6 months) than those with high CD206^+^ TAMs density (OS, 40.1 months, *p* = 0.003; RFS, 39.1 months, *p* = 0.021) (Figs. 3E and 3F). Collectively, these data indicated that CD86 and CD206 had a prognostic value in predicting survival and tumor recurrence of ICC patients.

### Combined analysis of CD86 and CD206 improves predictive value of ICC patient outcome

**Figure 4:**
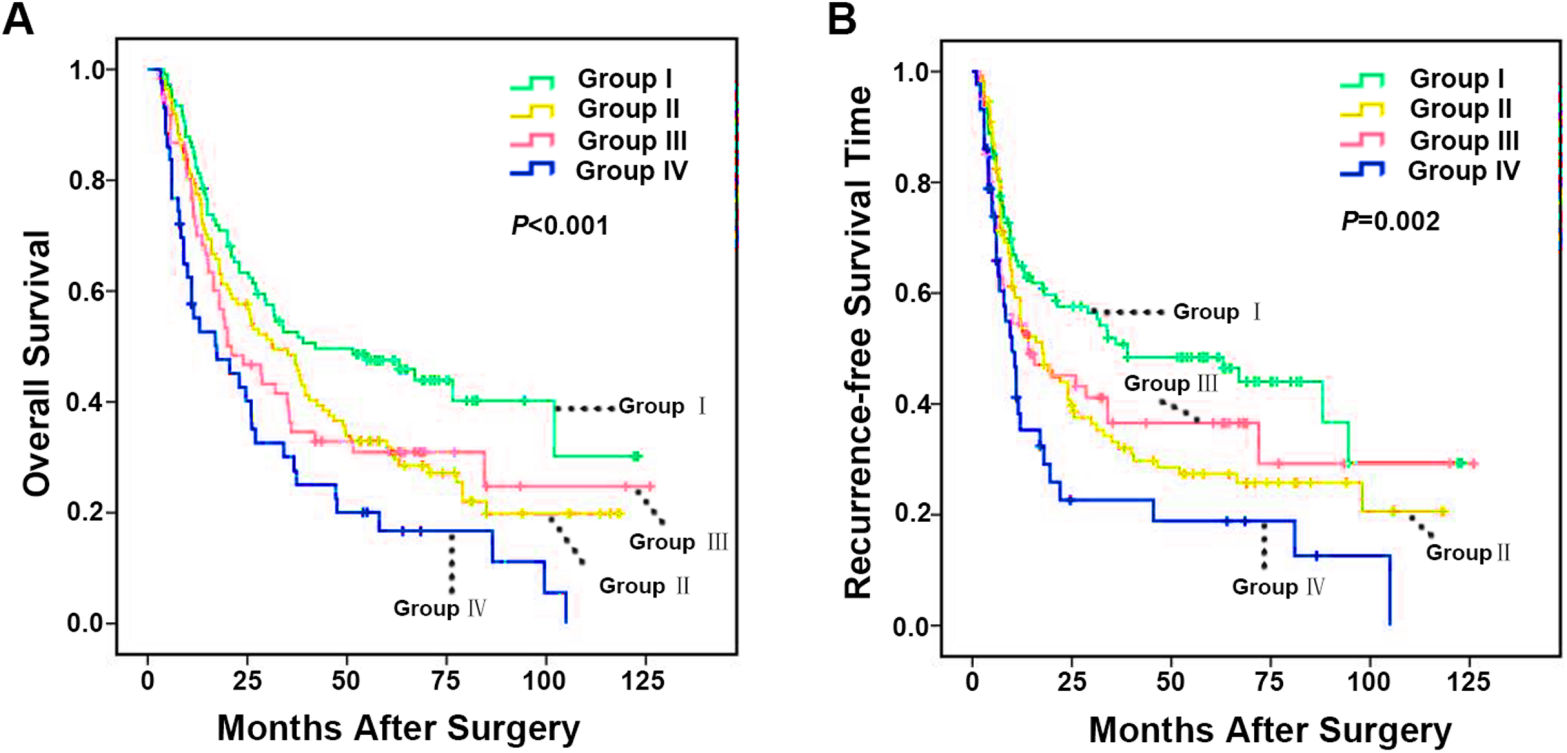
Kaplan–Meier analysis of OS and RFS in ICC according to integrated analysis of CD86^+^/CD206^+^ TAMs. (A) OS for integrated analysis of CD86^+^/CD206^+^ TAMs in 322 ICC patients and (B) RFS for integrated analysis of CD86^+^/CD206^+^ TAMs in 322 ICC patients. Group I, high immunostaining density of CD86^+^ but low CD206^+^ TAMs; Group II, both low immunostaining density of CD86^+^ and CD206^+^ TAMs; Group III, both high immunostaining density of CD86^+^ and CD206^+^ TAMs; Group IV, low immunostaining density of CD86^+^ but high CD206^+^ TAMs. *p* Values were calculated by log-rank test, and *p* < 0.05 was considered statistically significant. OS, Overall survival; RFS, recurrence-free survival.

Integrated analysis of CD86 and CD206 provided a more powerful prediction for ICC patient outcomes. Patients were classified into four subgroups based on intratumoral CD86^+^ and CD206^+^ TAMs number (I: CD86^high^/CD206^low^, II: CD86^low^/CD206^low^, III: CD86^high^/CD206^high^, IV: CD86^low^/CD206^high^). Subgroup comparisons showed that patients in the CD86^low^/CD206^high^ group had the worst prognosis and the greatest risk of tumor recurrence. Conversely, ICC patients in the CD86^high^/CD206^low^ group had the best prognosis (Figs. 4A and 4B). The median OS of Group I, II, III and IV were 62.8, 47.3, 48.7 and 31.3 months (*p* < 0.001), respectively. While the median RFS of Group I, II, III and IV were 58.9, 41.0, 48.5 and 26.7 months (*p* = 0.002) respectively. Taken together, these results evidently indicated that integrated analysis of immunomarkers CD86 and CD206 could serve as a more powerful predictor of prognosis in ICC patients.

As summarized in Tables 2 and 3, multivariable analyses revealed that apart from CA-199, lymph node metastasis and tumor multiplicity, CD86^+^/CD206^+^ TAMs model remained to be the independent prognostic indicator for both OS (*p* = 0.003) and RFS (*p* = 0.005). Collectively, these data suggested that integrated analysis of CD86^+^/CD206^+^ TAMs model is a valuable predictor for poor prognosis in ICC patients.

**Table 2:** Univariate and multivariate analysis of factors related to OS of ICC patients in the cohort (*n* = 322).

	OS			
	Univariate	Multivariate		
	*P*	HR	95% CI	*p*
Age, years (>51 vs. ≤51)	0.965			NA
Gender (male vs. female)	0.339			NA
Hepatitis history (positive vs. negative)	0.099			NA
CA199, U/ml (>36 vs. ≤36)	**<0.001**	**1.404**	**[1.069–1.843]**	**0.014**
Lymph node metastasis (yes vs. no)	**<0.001**	**1.974**	**[1.071–3.639]**	**0.029**
Liver cirrhosis (yes vs. no)	0.257			NA
Tumor size (cm) (>5 vs. ≤5)	**0.002**			NS
Tumor multiplicity (multiple vs. single)	**0.003**	**1.523**	**[1.121–2.069]**	**0.007**
Tumor encapsulation (none vs. complete)	0.134			NA
Tumor differentiation (poor vs. well)	0.581			NA
Vascular invasion (yes vs. no)	0.225			NA
TNM stage (III + IV vs. I + II)	**<0.001**			NS
CD86^+^/CD206^+^ TAMs predictive model^a^	**<0.001**			**0.003**
II vs. I	**0.028**	**1.433**	**[1.016–2.020]**	**0.040**
III vs. I	**0.043**			NS
IV vs. I	**<0.001**	**1.211**	**[1.039–1.411]**	**0.014**

**Notes:**

ICC, intrahepatic cholangiocarcinoma; OS, overall survival; TNM, tumor-node-metastasis; HR, hazard ratio; CI, confidential interval; NA, not applicable; NS, not significant. Univariate analysis was performed by Kaplan–Meier method (log-rank test). Multivariate analysis was calculated using the Cox multivariate proportional hazard regression model with stepwise manner.

Bold values indicate *P* < 0.05.

a Patients were divided into 4 groups based on their staining densities of CD86 and CD206 positive TAMs: group I, high expression of CD86 but low expression of CD206; group II, both low expressions; group III, both high expressions; group IV, low expression of CD86 but high expression of CD206.

**Table 3:** Univariate and multivariate analysis of factors related to RFS of ICC patients in the cohort (*n* = 322). ICC, intrahepatic cholangiocarcinoma; RFS, time interval from the date of surgery to recurrence; TNM, tumor-node-metastasis; HR, hazard ratio; CI, confidential interval; NA, not applicable; NS, not significant. Univariate analysis was performed by Kaplan–Meier method (log-rank test). Multivariate analysis was calculated using the Cox multivariate proportional hazard regression model with stepwise manner.

	RFS			
	Univariate	Multivariate		
	*P*	HR	95% CI	*p*
Age, years (>51 vs. ≤51)	0.353			NA
Gender (male vs. female)	0.572			NA
Hepatitis history (positive vs. negative)	0.365			NA
CA199, U/ml (>36 vs. ≤36)	**0.033**			NS
Lymph node metastasis (yes vs. no)	**<0.001**	**2.020**	**[1.401–2.914]**	**<0.001**
Liver cirrhosis (yes vs. no)	0.214			NA
Tumor size (cm) (>5 vs. ≤5)	**0.011**			NS
Tumor multiplicity (multiple vs. single)	**0.001**	**1.641**	**[1.193–2.257]**	**0.002**
Tumor encapsulation (none vs. complete)	0.108			NA
Tumor differentiation (poor vs. well)	0.876			NA
Vascular invasion (yes vs. no)	**0.026**			NS
TNM stage (III + IV vs. I + II)	**<0.001**			NS
CD86^+^/CD206^+^ TAMs predictive model^a^	**0.001**			**0.005**
II vs. I	**0.024**	**1.477**	**[1.042–2.094]**	**0.028**
III vs. I	0.108			NA
IV vs. I	**<0.001**	**1.181**	**[1.005–1.387]**	**0.043**

**Notes:**

Bold values indicate *P* < 0.05.

a Patients were divided into four groups based on their staining densities of CD86 and CD206 positive TAMs: group I, high expression of CD86 but low expression of CD206; group II, both low expressions; group III, both high expressions; group IV, low expression of CD86 but high expression of CD206.

### CD86^+^/CD206^+^ TAMs model predicts prognosis of carbohydrate antigen 199 negative ICC Patients

**Figure 5:**
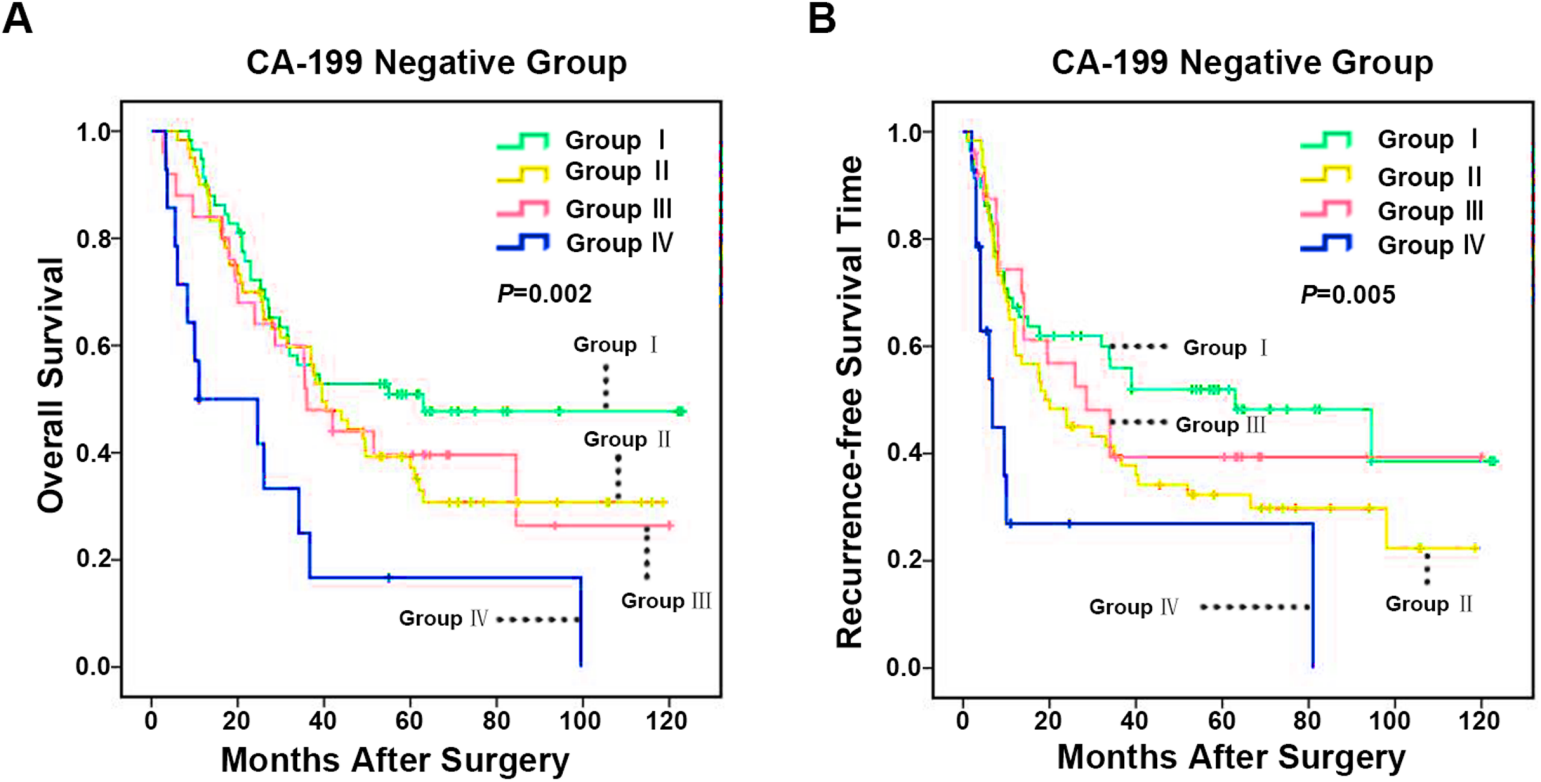
Kaplan–Meier analysis of OS and RFS in ICC with negative CA-199 based on CD86^+^/CD206^+^ TAMs. (A) ****OS for integrated analysis of CD86^+^/CD206^+^ TAMs in 157 ICC patients with negative CA-199 (CA-199 ≤ 36 U/mL) and (B) RFS for integrated analysis of CD86^+^/CD206^+^ TAMs in 157 ICC patients with negative CA-199. Group I, high immunostaining density of CD86^+^ but low CD206^+^ TAMs; Group II, both low immunostaining density of CD86^+^ and CD206^+^ TAMs; Group III, both high immunostaining density of CD86^+^ and CD206^+^ TAMs; Group IV, low immunostaining density of CD86^+^ but high CD206^+^ TAMs. *p* Values were calculated by log-rank test, and *p* < 0.05 was considered statistically significant. OS, Overall survival; RFS, recurrence-free survival.

Notably, several ICC prognostic studies indicated that preoperative serum CA-199 level was a significant prognostic indicator for ICC patients after hepatectomy (Bergquist et al., 2016; He et al., 2018; Juntermanns et al., 2010; Kondo et al., 2014; Zheng et al., 2017). In general, high serum tumor marker CA-199 level was markedly associated with advanced tumor staging, increased risk for tumor recurrence, and poor OS (Bergquist et al., 2016; Juntermanns et al., 2010; Yoo et al., 2015). Resected patients with CA19-9 elevation had similar peri-operative outcomes but decreased long-term survival (Bergquist et al., 2016). However, some ICC patients with low preoperative serum level of CA-199 was associated with rapid disease progression (He et al., 2018). Worse yet, a reliable tumor marker to distinguish the prognosis of CA-199 negative ICC patients remains limited. To investigate the prognostic effect of CD86^+^/CD206^+^ TAMs model in CA-199 negative patients (cut-off value 36 U/ml) (Chen et al., 2015), 163 patients from the 322 ICC patient cohort were enrolled. In this CA-199 negative cohort, patients in CD86^high^/CD206^low^ group had the best prognosis, while CD86^low^/CD206^high^ group patients had the worst prognosis. The median OS of Group I, II, III and IV were 72.2, 57.4, 56.9 and 30.1 months (*p* = 0.002), respectively (Fig. 5A) and the median RFS of Group I, II, III and IV were 65.2, 46.0, 56.7 and 25.9 months (*p* = 0.005), respectively (Fig. 5B).

### Construction and validation of the nomogram

**Figure 6:**
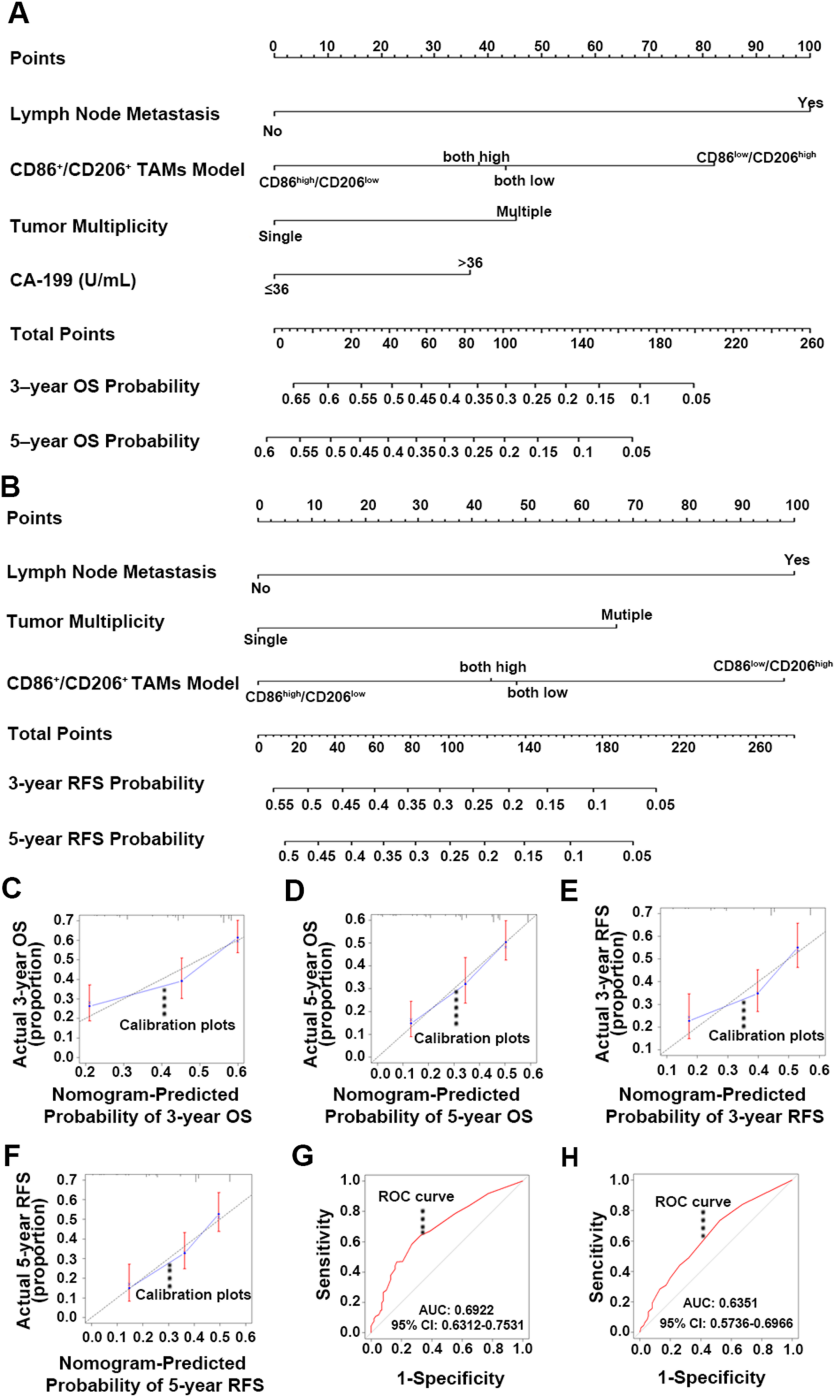
Nomogram for predicting 3- and 5-year OS and RFS, calibration plot and ROC curve analysis. (A) Nomogram for predicting ICC patients 3- and 5-year OS integrating lymph node metastasis, CD86^+^/CD206^+^ TAMs, tumor multiplicity and CA-199. (B) Nomogram for predicting ICC patients 3- and 5-year RFS integrating lymph node metastasis, CD86^+^/CD206^+^ TAMs, tumor multiplicity and CA-199. (C) Calibration plot for predicted and observed 3-year OS. (D) Calibration plot for predicted and observed 5 year OS. (E) Calibration plot for predicted and observed 3 year RFS. (F) Calibration plot for predicted and observed 5 year RFS. (G) ROC curve was performed to further evaluate the OS nomogram and (H) ROC curve was performed to further evaluate the RFS nomogram. OS, Overall survival; RFS, recurrence-free survival; ROC, receiver operating characteristic.

Independent prognostic indexes were identified through univariate analysis and forward stepwise (Bendel & Afifi, 1977 ) multivariable regression analysis. CA-199 (*p* = 0.014), lymph node metastasis (*p* = 0.029), tumor multiplicity (*p* = 0.007) and CD86^+^/CD206^+^ TAMs model (*p* = 0.003) were utilized to construct the OS nomogram (Fig. 6A). On the other hand, lymph node metastasis (*p* < 0.001), tumor multiplicity (*p* = 0.002) and CD86^+^/CD206^+^ TAMs model (*p* = 0.005) were selected to build the RFS nomogram (Fig. 6B). The summed-up points of each nomogram variable score indicated a more accurate hierarchical prediction of patient outcomes. Higher total points are corresponding to worse OS and RFS probability.

Internal validation of the nomograms was carried out by calibration plots with bootstrap sampling (*m* = 100, *n* = 1,000) (Harrell, Lee & Mark, 1996). The C-indexes for the OS and RFS nomograms were 0.649 and 0.635, respectively. Both calibration plots were closely related to the 45-degree line (Figs. 6C–6F). Additionally, the ROC was performed to further evaluate the nomograms (Linden, 2006; Robin et al., 2011). The AUC was 0.6922 (95% CI [0.6312–0.7531]) for the OS nomogram (Fig. 6G) and 0.6351 (95% CI [0.5736–0.6966]) for the RFS nomogram (Fig. 6H). These results illustrated that the predicted and observed survival probabilities were in good concordance, and the goodness-of-fit was favorable.

## Discussion

In this study, the prognostic significance of CD86^+^ and CD206^+^ TAMs in a large cohort of surgically resected ICC patients was investigated. Results demonstrated that high CD86^+^ and low CD206^+^ TAMs infiltration were significantly correlated with certain favorable tumor clinicopathologic features and better prognosis in ICC patients, when compared to low CD86^+^ and high CD206^+^ TAMs infiltration. Moreover, multivariable Cox regression analysis suggested that CD86^+^/CD206^+^ TAMs model was an independent prognostic indicator for ICC, especially in CA-199 negative patients. These results indicated that CD86^+^/CD206^+^ TAMs model may be a powerful prognostic indicator in ICC.

Although the mechanisms underlying ICC initiation and progression have not yet been elucidated, interactions between tumor cells and environmental signals are supposed to play an important role. Of the cellular components involved in the tumor microenvironment, TAMs infiltrates in most tumors, establishing a cross-talk bridge between tumor cells and immune microenvironment. Yet there is increasing evidence that subtypes of macrophages exhibit partly opposing properties in tumor initiation and development (Locatelli et al., 2016; Den Breems & Eftimie, 2016; Xu et al., 2014). It was worth noting that M1 and M2 polarization phenotypes represent extremes of a spectrum. In some cases, a mixed M1/M2 phenotype would be produced in response to different external signals (Biswas & Mantovani, 2010; Lawrence & Natoli, 2011). Moreover, there is always a dynamic switch between the different polarized statuses of TAMs in tumor microenvironment, which is depending on the local cytokine milieu. Under the circumstances of tumor progression, aberrant secretion of cytokines was often observed from tumor cells and their surrounding microenvironment, which educated macrophages to display trophic properties and switched to an immunosuppressive M2 polarization status (Senovilla et al., 2012).

CD68^+^ TAMs had no prognostic value in predicting the outcome of surgically treated ICC patients, which may be attributed to the inability of CD68 to distinguish between M1 and M2 subsets. This was consistent with previous studies in HCC, colon cancer and gastric cancer (Dong et al., 2016; Hernandez et al., 2014; Zhang et al., 2015). Therefore, CD86 and CD206 immunostaining were further examined to distinguish between M1 and M2 subtypes. As a result, CD86^high^/CD206^low^ subset exhibited a favorable prognosis in surgically treated ICC patients, while CD86^low^/CD206^high^ subset implied an inferior outcome. Interestingly, CD86^low^/CD206^low^ and CD86^high^/CD206^high^ profiles implying a mixed M1/M2 phenotype were associated with intermediate survival. Additionally, the prognostic power of combined CD86 and CD206 in CA-199 negative ICC patients was consistent with previous findings. Thus, macrophage immunomarkers CD86 and CD206 could be utilized as powerful indicators for rational treatment decision, especially in CA-199 negative patients. Furthermore, we found that the blood microvessel count was significantly higher in the CD206 high tissues compared with the CD206 low tissues, which indicated that M2 macrophages may stimulate angiogenesis. This is consistent with other previous studies (Bronkhorst et al., 2011; Xue et al., 2019). But there is no significant difference between the two groups in LMVD (*p* > 0.05) (Fig. S2C). The result is consistent with a previous study (Zhang et al., 2011) which showed a positive correlation between M2-polarized TAM count and peri-tumoral LMVD, but not intra-tumoral LMVD in patients with lung adenocarcinoma. Since our study only included intra-tumoral samples, further study will test more samples and include peri-tumoral LMVD.

In contrast to other prognostic markers, TAMs can also serve as therapeutic targets. Dong et al. (2017) identified that Fenretinide (4-HPR) could selectively inhibit M2 macrophage polarization through the inhibition of phosphorylation of STAT6, and in turn prevented the tumorigenesis of colorectal cancer. Recent studies have emphasized IFN-γ on its ability to switch immunosuppressive TAMs into immunostimulatory. Upon IFN-γ treatment, TAMs purified from ovarian cancer ascites recovered a M1 phenotype (IL-10^low^, IL-12^high^), increased expression of CD86 and decreased level of ILT3, enhanced the proliferation of CD4^+^ T lymphocytes and improved the cytotoxic properties of CD8^+^ T cells clone (Duluc et al., 2009). Depletion of TAMs using clodronate-loaded liposomes (clodrolip) enhanced the effect of sorafenib in metastatic liver cancer models by anti-metastatic and anti-angiogenic effects (Zhang et al., 2010). Moreover, TAMs depletion by GW2580, a selective pharmacologic inhibitor of CSF1R signaling, enhanced the anti-angiogenic and anti-tumor effects of VEGF/VEGFR2 antibodies in subcutaneous tumor models (Priceman et al., 2010). All these data implied that TAMs could be a potential target for tumor treatment. In spite of its potential importance in the prognostic and therapeutic value of cancer, the mechanism macrophage recruitment into tumors is still not fully understood. Further studies are needed to shed light on the exact mechanism.

## Conclusions

In conclusion, the present study demonstrated that the CD86^high^/CD206^low^ group representing M1 polarized status predicts a favorable outcome, while the CD86^low^/CD206^high^ group implying M2 polarized status suggests poor prognosis in surgically treated ICC patients, respectively. Combined analysis of CD86^+^/CD206^+^ TAMs provided a better prognostic value than individual analysis for ICC survival and recurrence, especially in CA-199 negative patients. Our findings provided a promising target for future investigation and intervention of ICC.

## Supplemental Information

10.7717/peerj.8458/supp-1 Supplemental Information 1 Immunofluorescence staining of Macrophages. (A) Representative photographs for CD68^+^ macrophages; (B) Representative photographs for CD86^+^ macrophages; (C) Representative photographs for CD206^+^ macrophages; (D) Nuclei were counterstained with DAPI; (E) Colocalization of CD68, CD86 and DAPI; (F) Colocalization of CD68, CD206 and DAPI; (G) Colocalization of CD206 and DAPI; (H) Colocalization of CD68, CD86, CD206 and DAPI. DAPI: 4’-6-diamidino-2-phenylindole. Magnification: × 400.

10.7717/peerj.8458/supp-2 Supplemental Information 2 M2-polarized Tumor-associated macrophages (M2-TAMs) and tumoral microvascular density (TMD) and lymphatic microvessel density (LMD) in ICC patients. (A) Immunohistochemical staining of M2-TAMs (staining by CD68 and CD206 antibodies) and tumoral MVD (staining by CD31 antibody) and LMVD (staining by LYVE-1 antibody). Case 4 showed a sample with high density of M2-TAMs, MVD and LMVD while case 11 showed a sample with low density of M2-TAMs, MVD and LMVD. (B) MVD was significantly increased in tumors with high density of M2-TAMs compared with those with low density of M2-TAMs. (C) There is no significant difference between the two groups with high density of M2-TAMs and with low density of M2-TAMs in LMVD. The MVD and LMVD were expressed as the mean ± SD. Magnification: × 200.

10.7717/peerj.8458/supp-3 Supplemental Information 3 Clinicopathologic characteristics and prognosis of ICC patients in the cohort (*n* = 322).

10.7717/peerj.8458/supp-4 Supplemental Information 4 Codebook of raw data.

## Additional Information and Declarations

### Data Availability

The following information was supplied regarding data availability: The raw data is available in the Supplemental Files.
