# Supplementary figures and images for: CD86+/CD206+ tumor-associated macrophages predict prognosis of patients with intrahepatic cholangiocarcinoma
Source: PeerJ. 2020 Jan 22;8:e8458. doi: 10.7717/peerj.8458 (PMC6982414; doi:10.7717/peerj.8458)

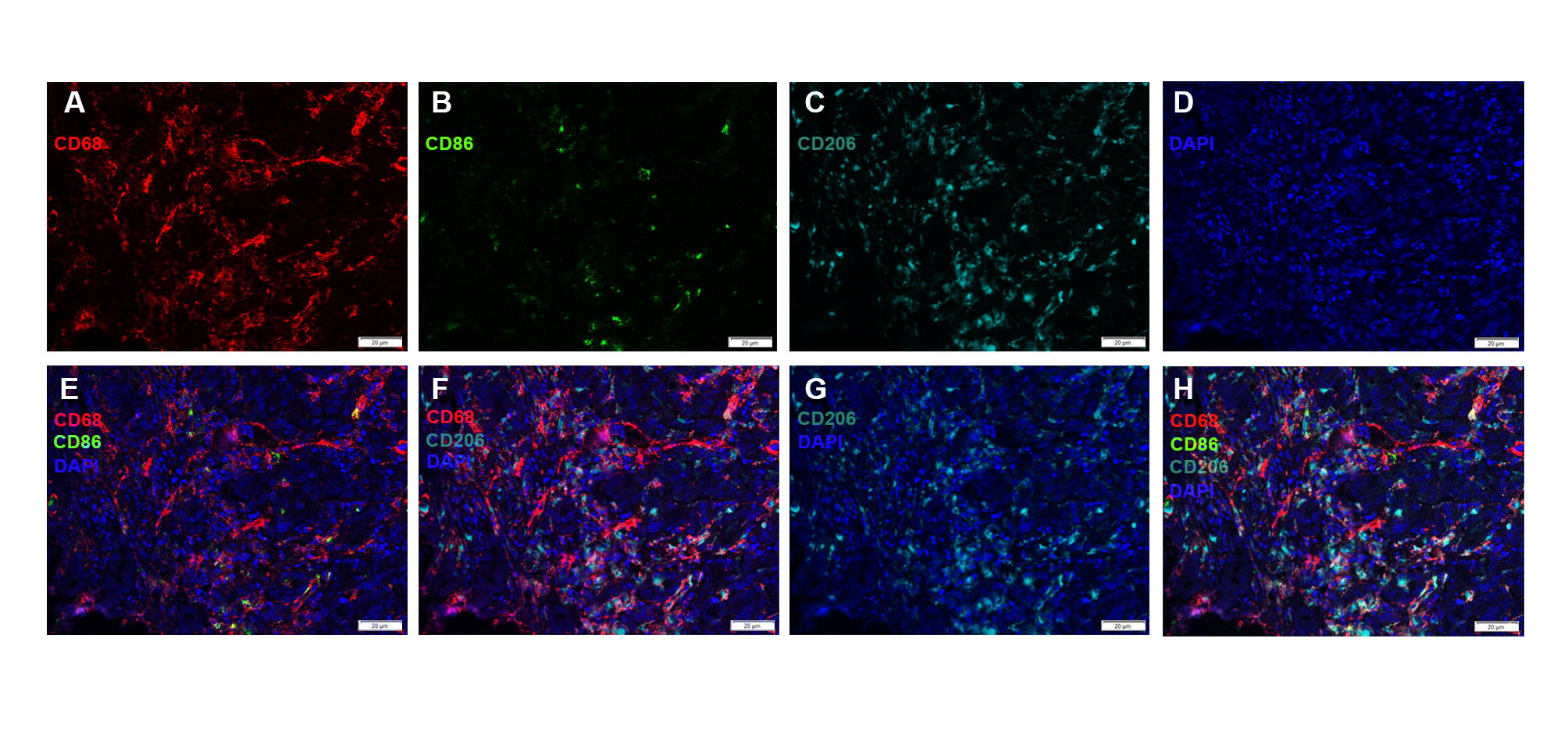

Supplement: Supplemental Information 1 — (A) Representative photographs for CD68+ macrophages; (B) Representative photographs for CD86+ macrophages; (C) Representative photographs for CD206+ macrophages; (D) Nuclei were counterstained with DAPI; (E) Colocalization of CD68, CD86 and DAPI; (F) Colocalization of CD68, CD206 and DAPI; (G) Colocalization of CD206 and DAPI; (H) Colocalization of CD68, CD86, CD206 and DAPI. DAPI: 4’-6-diamidino-2-phenylindole. Magnification: × 400. [file peerj-08-8458-s001.png]

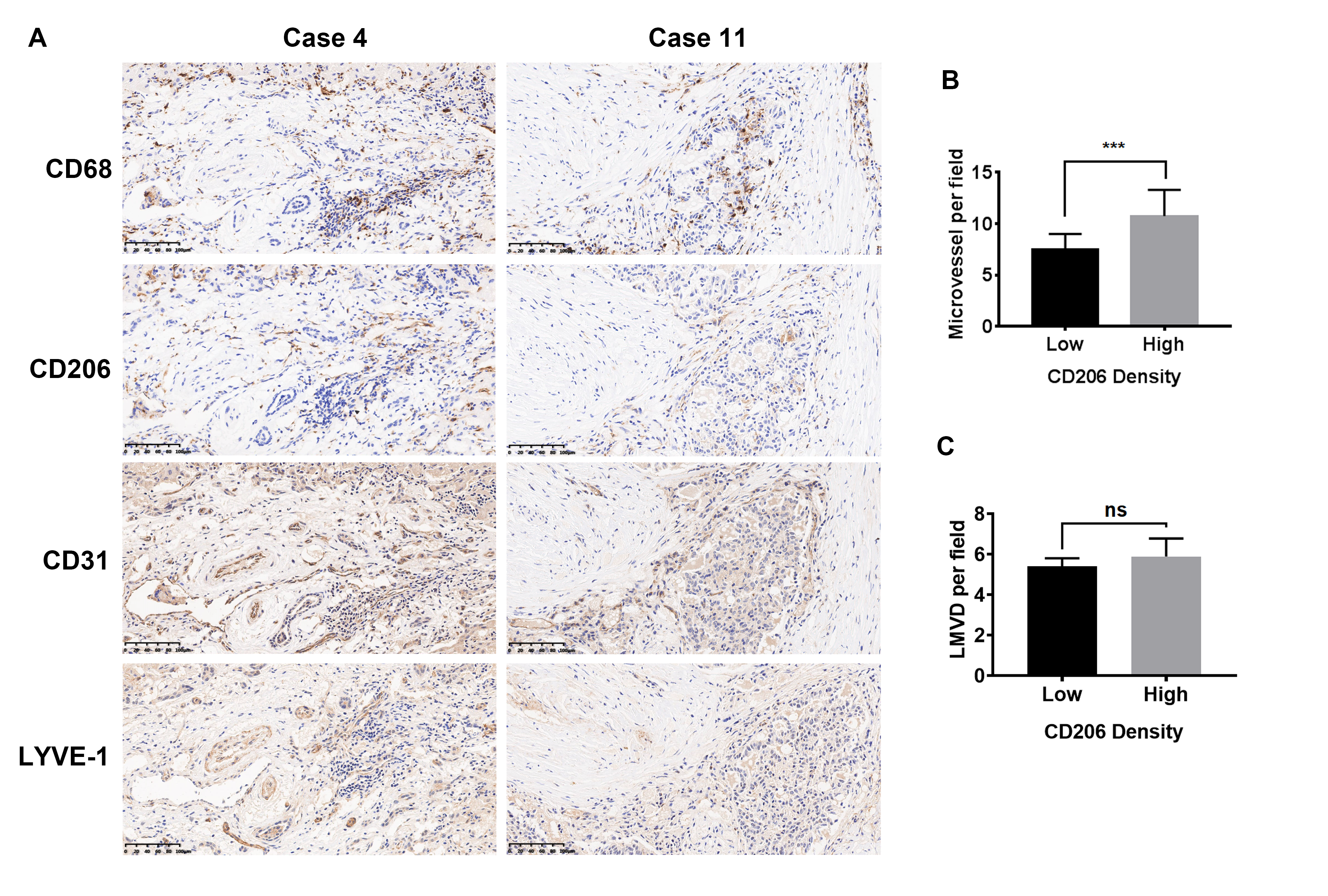

Supplement: Supplemental Information 2 — (A) Immunohistochemical staining of M2-TAMs (staining by CD68 and CD206 antibodies) and tumoral MVD (staining by CD31 antibody) and LMVD (staining by LYVE-1 antibody). Case 4 showed a sample with high density of M2-TAMs, MVD and LMVD while case 11 showed a sample with low density of M2-TAMs, MVD and LMVD. (B) MVD was significantly increased in tumors with high density of M2-TAMs compared with those with low density of M2-TAMs. (C) There is no significant difference between the two groups with high density of M2-TAMs and with low density of M2-TAMs in LMVD. The MVD and LMVD were expressed as the mean ± SD. Magnification: × 200. [file peerj-08-8458-s002.png]
